# Evaluation of high-risk type 1 diabetes HLA-DR and DQ haplotypes using three single nucleotide polymorphisms in a population from Southern Brazil

**DOI:** 10.1186/1758-5996-7-S1-A215

**Published:** 2015-11-11

**Authors:** Guilherme Coutinho Kullmann Duarte, Tais Silveira Assmann, Égina Marina Barbosa Martins, Mariana Lopes dos Santos, Luis Henrique Canani, Daisy Crispim

**Affiliations:** 1 Hospital de Clínicas de Porto Alegre, Porto Alegre, Brazil

## Background

Type 1 diabetes mellitus (T1D) accounts for ~10% of all diabetes cases, and it is caused by autoimmune destruction of pancreatic beta-cells, which leads to insulin deficiency and fates individuals to require insulin treatment to survive. The triggering of autoimmunity against beta-cells is caused by interaction between environmental and genetic risk factors. Among the several loci associated with T1D, the human leukocyte antigen (HLA) class II DR/DQ locus is the main genetic risk factor for T1D, accounting for 30-50% of genetic risk for this disease. Other genes have been associated with minor effects on T1D risk when compared with HLA, with different studies indicating that the effect of non-HLA polymorphisms on predisposition for T1D may be different according to HLA DR/DQ types. In this scenario, a recent study identified a minimum set of three polymorphisms (rs3104413, rs2854275, rs9273363) which can predict high-risk HLA-DR/DQ types relevant to T1D.

## Objective

To evaluate frequencies of high-risk T1D HLA-DR/DQ haplotypes in a Southern Brazilian population using a minimum set of HLA polymorphisms (rs3104413C/G, rs2854275A/C and rs9273363A/C).

## Materials and methods

We analyzed 387 T1D patients (cases) and 375 healthy blood-donor subjects (controls). The local ethics committee approved the protocol, and all patients signed an informed consent form. Polymorphisms of interest were genotyped by allelic discrimination – RT-PCR technique using TaqMan MGB probes (Life Technologies). Haplotype combinations of the three analyzed polymorphisms were used for defining the HLA types relevant to T1D (Nguyen et al*), distinguishing the highest-risk DR4-DQ8 and DR3/4-DQ types.

## Results

Minor alleles frequencies of rs3104413, rs2854275, rs9273363 were increased in T1D patients as compared to non-diabetic subjects (rs3104413C: 44.4% vs. 11.2%; rs2854275A: 12.9% vs. 1.9%; rs9273363A: 43.9% vs. 9.9%; all P <0.0001).The frequency of high-risk DR4-DQ8 type was 66.7% in T1D cases and 15.3% in controls (OR=11.059, 95%CI 6.68-18.29; P <0.0001).The high-risk DR3/4-DQ8 heterozygous haplotype was observed in only one T1D patients and in none control subject.

## Conclusion

As expected, the high-risk HLA-DR4/DQ8 haplotype is associated with increased risk for T1D in our population. The genetic risk of non-HLA genes on T1D in Southern Brazil can now be correct for different high-risk HLA-DR/DQ types.
